# Diffuse pulmonary lymphangiomatosis as a differential diagnosis of anterior mediastinal mass

**DOI:** 10.1093/jscr/rjae577

**Published:** 2024-09-12

**Authors:** Diego Salcedo Miranda, Jorge Roberto Galvis, Luis Jaime Téllez Rodríguez, Juan Carlos Garzón Ramírez, Julián Ariza Traslaviña

**Affiliations:** Thoracic Surgery, National Cancer Institute – El Bosque University, Bogotá, 111511, Colombia; General Surgery, El Bosque University, Bogotá, 110121, Colombia; Department of Thoracic Surgery, Fundación Cardioinfantil, Bogotá, 111321, Colombia; Department of Thoracic Surgery, Fundación Cardioinfantil, Bogotá, 111321, Colombia; Department of Thoracic Surgery, Fundación Cardioinfantil, Bogotá, 111321, Colombia

**Keywords:** diffuse pulmonary lymphangiomatosis, surgical intervention, septal thickening, radiology, pathology

## Abstract

Diffuse pulmonary lymphangiomatosis (DLP) is an extremely rare silent disease, characterized by proliferation and thickening of abnormal pulmonary, pleural, and mediastinal soft tissue lymphatic channels. Its clinical presentation is nonspecific symptoms such as cough, dyspnea, and hemoptysis. Tomographic findings for DLP include thickening of the interlobular septa and peribronchovascular interstitium and ground glass opacities. Nevertheless, the anterior mediastinal mass, associated with thickening of interlobular septa and peribronchovascular interstitial, ground glass opacities, pleural effusion, diffuse infiltration of the mediastinum and pleural thickening in a patient with lymphangiomas, DLP should be suspected as a differential diagnosis.

## Introduction

The most common anterior mediastinal masses are: thymoma, teratoma, endothoracic goiter, and lymphoma [1]. However, we must keep in mind other pathologies that could occur, such as an anterior mediastinal mass secondary to diffuse pulmonary lymphangiomatosis (DLP).

The computed tomography (CT) imaging findings for DLP are thickening of interlobular septa and peribronchovascular interstitium, ground glass opacities, pleural effusion, diffuse infiltration of the mediastinum, and pleural thickening [2]; but it is not associated imaging with an anterior mediastinal mass, so if the diagnosis is suspected, magnetic resonance imaging should be considered, in which pulmonary and mediastinal cystic lesions are evident, associated with pulmonary linear opacities [3, 4], the presence of diffuse pulmonary lymphangiomatosis would be suspected. Cystic lesions are hypointense on T1W1 and hyperintense on T2W1 and do not enhance contrast or very little peripheral contrast enhancement is evident [5].

DLP involves both lungs and does not present extrathoracic lymphatic involvement. The average age of diagnosis of these patients is 48 years of age and 40% are men [2]. The pathogenesis of this disease remains to be elucidated. The main cause of this disease is caused by congenital factors and acquired diseases [6]. Once the disease is suspected, the diagnosis is confirmed by taking a lung or pleural biopsy by thoracoscopy and/or mediastinal biopsy with histological diagnosis. Its medical management is with immunosuppressants, biological compounds, vascular endothelial growth factors, among others to control symptoms and control the progression of the disease [7].

Therefore, we present the case of a patient with a history of cutaneous lymphangiomas, with an anterior mediastinal mass with thickening of interlobular septa and peribronchovascular interstitium, which was confirmed as DLP with pulmonary wedge by thoracoscopy and histology, which must be within the differential diagnosis of anterior mediastinal mass.

## Case report

**Figure 1:**
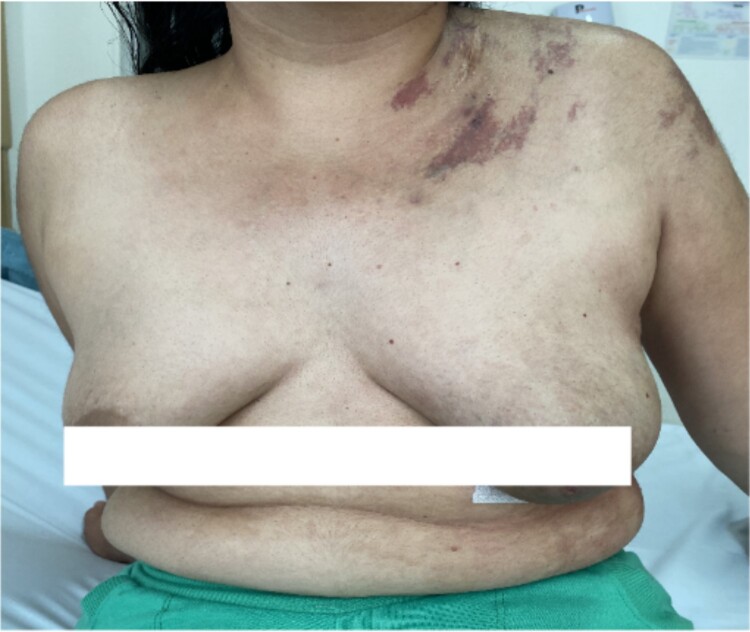
Cutaneous vascular lesions.

We present the case of a 29-year-old woman with a history of cutaneous lymphangiomas, consulted for a 3-month history of clinical symptoms characterized by hemoptysis, nocturnal diaphoresis, 6 kg weight loss, and in the last 10 days she presented dyspnea and chest pain. On physical examination, cutaneous vascular lesions in the left cervical area, anterior wall of the left hemithorax and deltoid area (Fig. 1), without palpable lymphadenopathy.

**Figure 2:**
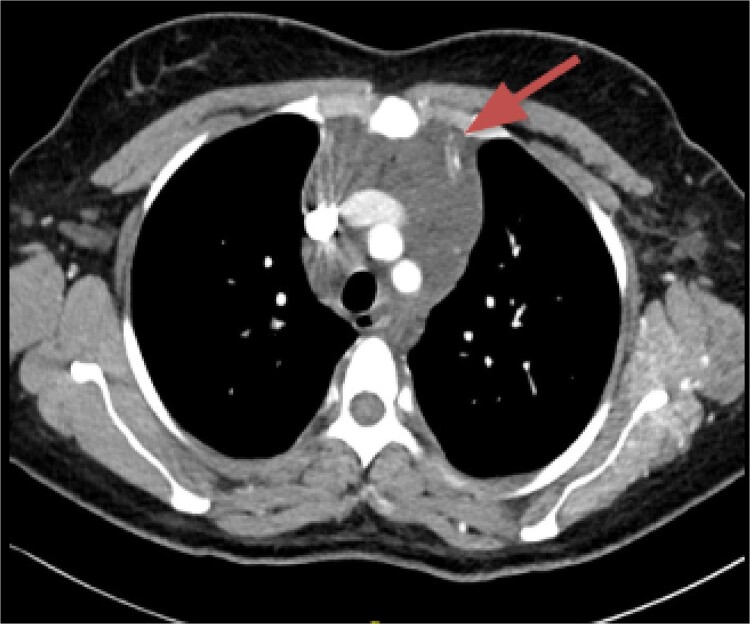
Mediastinal mass.

**Figure 3:**
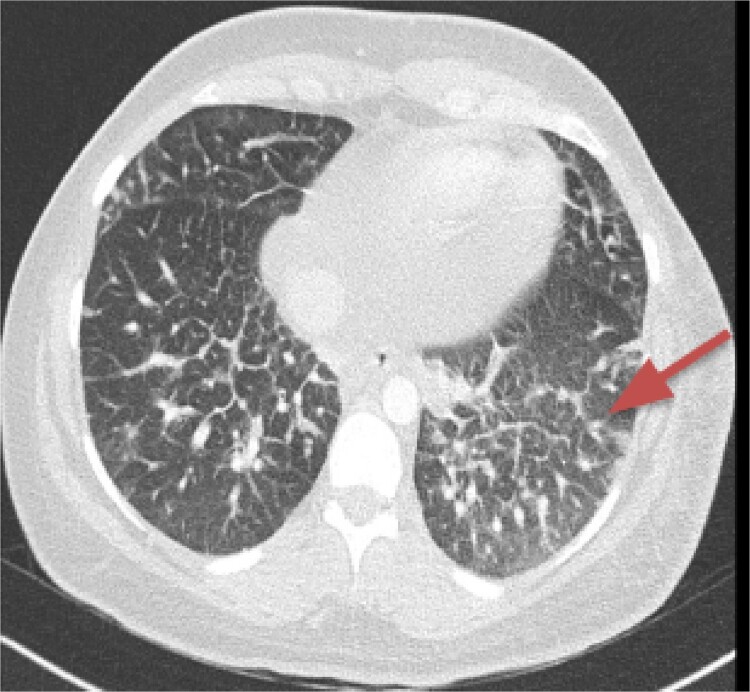
Thickening of interlobular septa in both lungs.

**Figure 4:**
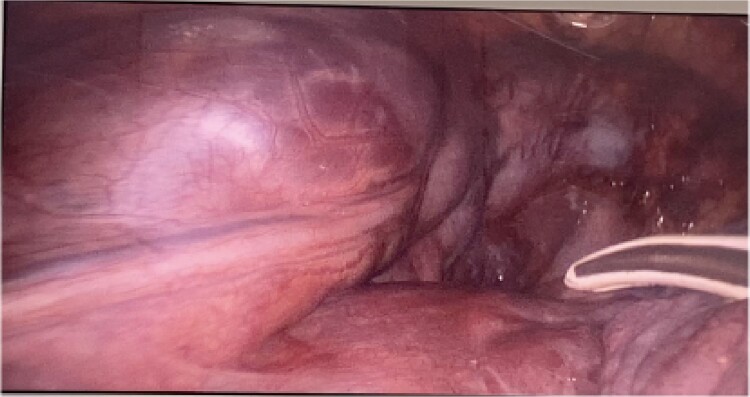
Mediastinal mass with cystic characteristics.

CT imaging, an anterior mediastinal mass was evident (Fig. 2), associated with the presence of thickening of interlobular septa in both lungs (Fig. 3). Surgery is carried out with diagnostic intent with suspicion of lymphoma vs DLP. A mass with cystic characteristics was found, thickening of interlobular septa and lymphatic channels that extended from the lung parenchyma to the mediastinum through the peribronchovascular space, dark serohematic pleural effusion without pleural lesions (Fig. 4).

**Figure 5:**
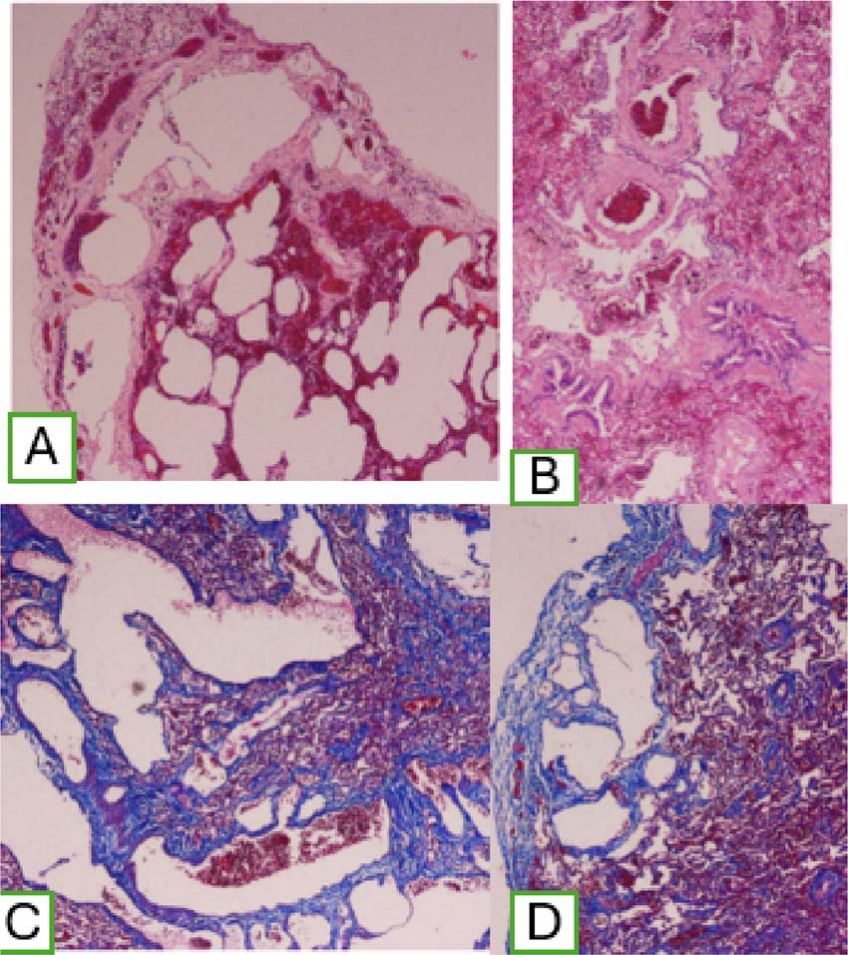
(A) Dilatation of subpleural lymphatic vessels; (B) peribronchovascular compromise; and (C) positive Masson staining.

A two pulmonary wedge was performed. In the microscopic findings, lung parenchyma with the presence of dilated lymphatic vessels, subpleural location, that extend through the interlobular septa, of varying sizes, with peribronchovascular involvement, with positive marking for Masson stain. Histological examination showed and confirmed DLP (Fig. 5).

## Discussion

DLP is extremely rare in adults [8]. The symptoms of this disease are wheezing, dyspnea, hemoptysis, chyloptysis; more common in adolescence [9]. It is thought that when chyloptysis occurs it is secondary to lymphatic flow stasis, generating reflux to the tracheobronchial tree. It may also present chylopericardium, chylous ascites, protein-losing enteropathy, lymphopenia, among others [10]. It can be associated with other pathologies, for example, cirrhosis and hepatic encephalopathy, or with skin lesions with or without lymphadenopathy, as evidenced in our case [6].

The tomographic findings of DLP are interlobular septal thickening (70%), peribronchovascular interstitial thickening, ground glass opacities (80%), pleural effusion (40%), diffuse mediastinal infiltration (90%), and pleural thickening [2, 4], but no mediastinal mass is documented on CT. The radiological differential diagnoses of this disease are pulmonary edema, pulmonary veno-occlusive disease, pulmonary lymphangitic carcinomatosis, sarcoidosis, amyloidosis, primary pulmonary lymphoma, Erdheim-Chester disease, but these are not associated with mediastinal abnormalities [2].

Given the above, it is important to use other diagnostic methods that allow obtaining findings with greater precision; for example, using multidetector CT images after direct lymphangiography, obtaining findings such as large accumulation of contrast in the mediastinum, in bronchovascular septa and in pleural and extrapleural soft tissues; suggesting that the abnormal soft tissue thickening was caused by dilation of lymphatic vessels or retrograde lymph flow associated with edema [11]. Magnetic resonance imaging is also important, which is a precise and safe, non-invasive study [12], in which the lymphatic vessels can be clearly differentiated in the T2 sequence, given that lymphatic flow is very slow in lymphangiomatous malformations [13, 14].

**Figure 6:**
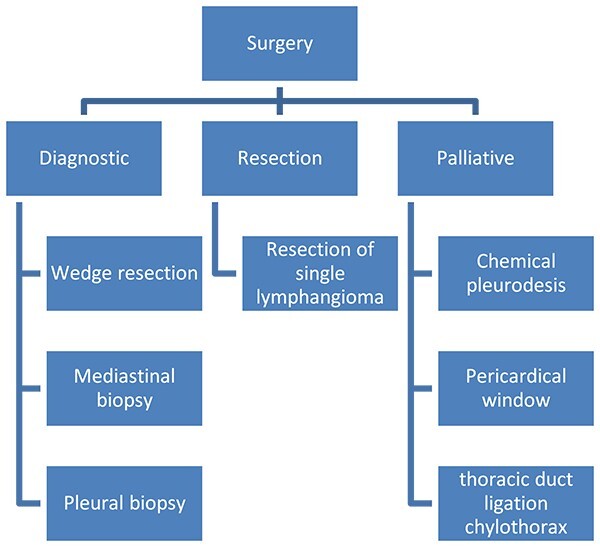
Surgery in diffuse pulmonary lymphangiomatosis.

Surgery in DLP has three objectives (Fig. 6): (i) Diagnostic surgery: The definitive diagnosis is obtained by histopathology; Minimally invasive mediastinal biopsy or thoracoscopy lung biopsy [7]. Characteristic of these is the proliferation and increase of lymphatic channels that have a connection between the mediastinum and the lungs [15]. Associated with this, hemosiderin–laden macrophages can be found adjacent to the lung parenchyma [4].

(ii) Resection surgery: Regarding surgical treatment consider only for small or localized lymphangiomas, it is sometimes challenging to differentiate between diseased and healthy lymphatic tissue. It is also very important to be able to completely resect the lesions, given that, if there is residual tissue, it can proliferate and cause the symptoms to appear again; this is technically challenging, given the proximity of diseased tissue to vital structures and organs [4]. Resections can be performed by thoracoscopy or thoracotomy.

(iii) Palliative surgery: relieve symptoms, with parietal pleurectomy, pleurodesis, and ligation of the thoracic duct, to reduce recurrent pleural effusion [4] (Fig. 6).

There is no specific treatment for DLP, nevertheless, the anterior mediastinal mass, associated with thickening of interlobular septa and peribronchovascular interstitial, ground glass opacities, pleural effusion, diffuse infiltration of the mediastinum, and pleural thickening in a patient with lymphangiomas, DLP should be suspected as a differential diagnosis.

## References

[1] Carter BW , Marom EM, Detterbeck FC. Approaching the patient with an anterior mediastinal mass: a guide for clinicians. J Thorac Oncol 2014;9:S102–9. https://doi.org/10.1097/JTO.0000000000000294. 25396306

[2] Zhang S , Zhong D, Zhao L, et al. Diffuse pulmonary lymphangiomatosis involving lungs and mediastinal soft tissue. Am J Med Sci 2022;364:118–23. https://doi.org/10.1016/j.amjms.2022.03.015. 35405139

[3] Mehrnahad M , Kord A, Rezaei Z, et al. Late diagnosis of generalized lymphangiomatosis in a woman presenting with respiratory distress. Radiol Case Rep 2020;15:1189–93. https://doi.org/10.1016/j.radcr.2020.05.021. 32550956 PMC7292890

[4] Luisi F , Torre O, Harari S. Thoracic involvement in generalised lymphatic anomaly (or lymphangiomatosis). Eur Respir Rev 2016;25:170–7. https://doi.org/10.1183/16000617.0018-2016. 27246594 PMC9487238

[5] Uribe R , Isaza S, Prada V, et al. Lymphangiomatosis in a 14-year-old female presenting with chylothorax and multiple cystic lesions. Radiol Case Rep 2018;13:782–7. https://doi.org/10.1016/j.radcr.2018.05.002. 30002781 PMC6041379

[6] Yu W , Mi L, Cong J, et al. Diffuse pulmonary lymphangiomatosis: a rare case report in an adult. Medicine (Baltimore) 2019;98:e17349. https://doi.org/10.1097/MD.0000000000017349. 31651839 PMC6824776

[7] Dimiene I , Bieksiene K, Zaveckiene J, et al. Effective initial treatment of diffuse pulmonary Lymphangiomatosis with Sirolimus and propranolol: a case report. Medicina (Kaunas) 2021;57:1308. https://doi.org/10.3390/medicina57121308. 34946253 PMC8706407

[8] Itkin M , McCormack FX. Nonmalignant adult thoracic lymphatic disorders. Clin Chest Med 2016;37:409–20. https://doi.org/10.1016/j.ccm.2016.04.004. 27514588

[9] El Hajj L , Mazières J, Rouquette I, et al. Diagnostic value of bronchoscopy, CT and transbronchial biopsies in diffuse pulmonary lymphangiomatosis: case report and review of the literature. Clin Radiol 2005;60:921–5. https://doi.org/10.1016/j.crad.2005.03.006. 16039928

[10] Faul JL , Berry GJ, Colby TV, et al. Thoracic lymphangiomas, lymphangiectasis, lymphangiomatosis, and lymphatic dysplasia syndrome. Am J Respir Crit Care Med 2000;161:1037–46. https://doi.org/10.1164/ajrccm.161.3.9904056. 10712360

[11] Sun X , Shen W, Xia S, et al. Diffuse pulmonary Lymphangiomatosis: MDCT findings after direct lymphangiography. AJR Am J Roentgenol 2017;208:300–5. https://doi.org/10.2214/AJR.16.16589. 27845836

[12] Lohrmann C , Foeldi E, Langer M. Assessment of the lymphatic system in patients with diffuse lymphangiomatosis by magnetic resonance imaging. Eur J Radiol 2011;80:576–81. https://doi.org/10.1016/j.ejrad.2009.10.021. 19913379

[13] Lu Q , Xu J, Liu N. Chronic lower extremity lymphedema: a comparative study of high-resolution interstitial MR lymphangiography and heavily T2-weighted MRI. Eur J Radiol 2010;73:365–73. https://doi.org/10.1016/j.ejrad.2008.10.041. 19108973

[14] Nagano N , Izumi S, Katsuno T, et al. A case of diffuse pulmonary lymphangiomatosis with a venous anomaly presenting with acute respiratory failure and hemoptysis. Respir Med Case Rep 2020;31:101243. https://doi.org/10.1016/j.rmcr.2020.101243. 33088708 PMC7567044

[15] Tazelaar HD , Kerr D, Yousem SA, et al. Diffuse pulmonary lymphangiomatosis. Hum Pathol 1993;24:1313–22. https://doi.org/10.1016/0046-8177(93)90265-I. 8276379

